# What is Qualitative in Qualitative Research

**DOI:** 10.1007/s11133-019-9413-7

**Published:** 2019-02-27

**Authors:** Patrik Aspers, Ugo Corte

**Affiliations:** 1 Department of Sociology, Uppsala University, Uppsala, Sweden; 2 Seminar for Sociology, Universität St. Gallen, St. Gallen, Switzerland; 3 Department of Media and Social Sciences, University of Stavanger, Stavanger, Norway

**Keywords:** Qualitative research, Methods, Epistemology, Philosophy of science, Phenomenology

## Abstract

What is qualitative research? If we look for a precise definition of qualitative research, and specifically for one that addresses its distinctive feature of being “qualitative,” the literature is meager. In this article we systematically search, identify and analyze a sample of 89 sources using or attempting to define the term “qualitative.” Then, drawing on ideas we find scattered across existing work, and based on Becker’s classic study of marijuana consumption, we formulate and illustrate a definition that tries to capture its core elements. We define qualitative research as an iterative process in which improved understanding to the scientific community is achieved by making new significant distinctions resulting from getting closer to the phenomenon studied. This formulation is developed as a tool to help improve research designs while stressing that a qualitative dimension is present in quantitative work as well. Additionally, it can facilitate teaching, communication between researchers, diminish the gap between qualitative and quantitative researchers, help to address critiques of qualitative methods, and be used as a standard of evaluation of qualitative research.

If we assume that there is something called qualitative research, what exactly is this qualitative feature? And how could we evaluate qualitative research as good or not? Is it fundamentally different from quantitative research? In practice, most active qualitative researchers working with empirical material intuitively know what is involved in doing qualitative research, yet perhaps surprisingly, a clear definition addressing its key feature is still missing.

To address the question of what is qualitative we turn to the accounts of “qualitative research” in textbooks and also in empirical work. In his classic, explorative, interview study of deviance Howard Becker (1963) asks ‘How does one become a marijuana user?’ In contrast to pre-dispositional and psychological-individualistic theories of deviant behavior, Becker’s inherently social explanation contends that becoming a user of this substance is the result of a three-phase sequential learning process. First, potential users need to learn how to smoke it properly to produce the “correct” effects. If not, they are likely to stop experimenting with it. Second, they need to discover the effects associated with it; in other words, to get “high,” individuals not only have to experience what the drug does, but also to become aware that those sensations are related to using it. Third, they require learning to savor the feelings related to its consumption – to develop an acquired taste. Becker, who played music himself, gets close to the phenomenon by observing, taking part, and by talking to people consuming the drug: “half of the fifty interviews were conducted with musicians, the other half covered a wide range of people, including laborers, machinists, and people in the professions” (Becker 1963:56).

Another central aspect derived through the common-to-all-research interplay between induction and deduction (Becker 2017), is that during the course of his research Becker adds scientifically meaningful new distinctions in the form of three phases—distinctions, or findings if you will, that strongly affect the course of his research: its focus, the material that he collects, and which eventually impact his findings. Each phase typically unfolds through social interaction, and often with input from experienced users in “a sequence of social experiences during which the person acquires a conception of the meaning of the behavior, and perceptions and judgments of objects and situations, all of which make the activity possible and desirable” (Becker 1963:235). In this study the increased understanding of smoking dope is a result of a combination of the meaning of the actors, and the conceptual distinctions that Becker introduces based on the views expressed by his respondents. Understanding is the result of research and is due to an *iterative process* in which data, concepts and evidence are connected with one another (Becker 2017).

Indeed, there are many definitions of qualitative research, but if we look for a definition that addresses its distinctive feature of being “qualitative,” the literature across the broad field of social science is meager. The main reason behind this article lies in the paradox, which, to put it bluntly, is that researchers act as if they know what it is, but they cannot formulate a coherent definition. Sociologists and others will of course continue to conduct good studies that show the relevance and value of qualitative research addressing scientific and practical problems in society. However, our paper is grounded in the idea that providing a clear definition will help us improve the work that we do. Among researchers who practice qualitative research there is clearly much knowledge. We suggest that a definition makes this knowledge more explicit. If the first rationale for writing this paper refers to the “internal” aim of improving qualitative research, the second refers to the increased “external” pressure that especially many qualitative researchers feel; pressure that comes both from society as well as from other scientific approaches. There is a strong core in qualitative research, and leading researchers tend to agree on what it is and how it is done. Our critique is not directed at the practice of qualitative research, but we do claim that the type of systematic work we do has not yet been done, and that it is useful to improve the field and its status in relation to quantitative research.

The literature on the “internal” aim of improving, or at least clarifying qualitative research is large, and we do not claim to be the first to notice the vagueness of the term “qualitative” (Strauss and Corbin 1998). Also, others have noted that there is no single definition of it (Long and Godfrey 2004:182), that there are many different views on qualitative research (Denzin and Lincoln 2003:11; Jovanović 2011:3), and that more generally, we need to define its meaning (Best 2004:54). Strauss and Corbin (1998), for example, as well as Nelson et al. (1992:2 cited in Denzin and Lincoln 2003:11), and Flick (2007:ix–x), have recognized that the term is problematic: “Actually, the term ‘qualitative research’ is confusing because it can mean different things to different people” (Strauss and Corbin 1998:10–11). Hammersley has discussed the possibility of addressing the problem, but states that “the task of providing an account of the distinctive features of qualitative research is far from straightforward” (2013:2). This confusion, as he has recently further argued (Hammersley 2018), is also salient in relation to ethnography where different philosophical and methodological approaches lead to a lack of agreement about what it means.

Others (e.g. Hammersley 2018; Fine and Hancock 2017) have also identified the treat to qualitative research that comes from external forces, seen from the point of view of “qualitative research.” This threat can be further divided into that which comes from inside academia, such as the critique voiced by “quantitative research” and outside of academia, including, for example, New Public Management. Hammersley (2018), zooming in on one type of qualitative research, ethnography, has argued that it is under treat. Similarly to Fine (2003), and before him Gans (1999), he writes that ethnography’ has acquired a range of meanings, and comes in many different versions, these often reflecting sharply divergent epistemological orientations. And already more than twenty years ago while reviewing Denzin and Lincoln’ s *Handbook of Qualitative Methods* Fine argued:

While this increasing centrality [of qualitative research] might lead one to believe that consensual standards have developed, this belief would be misleading. As the methodology becomes more widely accepted, querulous challengers have raised fundamental questions that collectively have undercut the traditional models of how qualitative research is to be fashioned and presented (1995:417).

According to Hammersley, there are today “serious treats to the practice of ethnographic work, on almost any definition” (2018:1). He lists five external treats: (1) that social research must be accountable and able to show its impact on society; (2) the current emphasis on “big data” and the emphasis on quantitative data and evidence; (3) the labor market pressure in academia that leaves less time for fieldwork (see also Fine and Hancock 2017); (4) problems of access to fields; and (5) the increased ethical scrutiny of projects, to which ethnography is particularly exposed. Hammersley discusses some more or less insufficient existing definitions of ethnography.

The current situation, as Hammersley and others note—and in relation not only to ethnography but also qualitative research in general, and as our empirical study shows—is not just unsatisfactory, it may even be harmful for the entire field of qualitative research, and does not help social science at large. We suggest that the lack of clarity of qualitative research is a real problem that must be addressed.

## Towards a Definition of Qualitative Research

Seen in an historical light, what is today called qualitative, or sometimes ethnographic, interpretative research – or a number of other terms – has more or less always existed. At the time the founders of sociology – Simmel, Weber, Durkheim and, before them, Marx – were writing, and during the era of the *Methodenstreit* (“dispute about methods”) in which the German historical school emphasized scientific methods (cf. Swedberg 1990), we can at least speak of qualitative forerunners.

Perhaps the most extended discussion of what later became known as qualitative methods in a classic work is Bronisław Malinowski’s (1922) *Argonauts in the Western Pacific*, although even this study does not explicitly address the meaning of “qualitative.” In Weber’s ([1921–-22] 1978) work we find a tension between scientific explanations that are based on observation and quantification and interpretative research (see also Lazarsfeld and Barton 1982).

If we look through major sociology journals like the *American Sociological Review*, *American Journal of Sociology*, or *Social Forces* we will not find the term qualitative sociology before the 1970s. And certainly before then much of what we consider qualitative classics in sociology, like Becker’ study (1963), had already been produced. Indeed, the Chicago School often combined qualitative and quantitative data within the same study (Fine 1995). Our point being that before a disciplinary self-awareness the term quantitative preceded qualitative, and the articulation of the former was a political move to claim scientific status (Denzin and Lincoln 2005). In the US the World War II seem to have sparked a critique of sociological work, including “qualitative work,” that did not follow the scientific canon (Rawls 2018), which was underpinned by a scientifically oriented and value free philosophy of science. As a result the attempts and practice of integrating qualitative and quantitative sociology at Chicago lost ground to sociology that was more oriented to surveys and quantitative work at Columbia under Merton-Lazarsfeld. The quantitative tradition was also able to present textbooks (Lundberg 1951) that facilitated the use this approach and its “methods.” The practices of the qualitative tradition, by and large, remained tacit or was part of the mentoring transferred from the renowned masters to their students.

This glimpse into history leads us back to the lack of a coherent account condensed in a definition of qualitative research. Many of the attempts to define the term do not meet the requirements of a proper definition: A definition should be clear, avoid tautology, demarcate its domain in relation to the environment, and ideally only use words in its *definiens* that themselves are not in need of definition (Hempel 1966). A definition can enhance precision and thus clarity by identifying the core of the phenomenon. Preferably, a definition should be short. The typical definition we have found, however, is an ostensive definition, which indicates what qualitative research is *about* without informing us about *what it actually is*:

Qualitative research is multimethod in focus, involving an interpretative, naturalistic approach to its subject matter. This means that qualitative researchers study things in their natural settings, attempting to make sense of, or interpret, phenomena in terms of the meanings people bring to them. Qualitative research involves the studied use and collection of a variety of empirical materials – case study, personal experience, introspective, life story, interview, observational, historical, interactional, and visual texts – that describe routine and problematic moments and meanings in individuals’ lives. (Denzin and Lincoln 2005:2)

Flick claims that the label “qualitative research” is indeed used as an umbrella for a number of approaches (2007:2–4; 2002:6), and it is not difficult to identify research fitting this designation. Moreover, whatever it is, it has grown dramatically over the past five decades. In addition, courses have been developed, methods have flourished, arguments about its future have been advanced (for example, Denzin and Lincoln 1994) and criticized (for example, Snow and Morrill 1995), and dedicated journals and books have mushroomed. Most social scientists have a clear idea of research and how it differs from journalism, politics and other activities. But the question of what is *qualitative* in qualitative research is either eluded or eschewed.

We maintain that this lacuna hinders systematic knowledge production based on qualitative research. Paul Lazarsfeld noted the lack of “codification” as early as 1955 when he reviewed 100 qualitative studies in order to offer a codification of the practices (Lazarsfeld and Barton 1982:239). Since then many texts on “qualitative research” and its methods have been published, including recent attempts (Goertz and Mahoney 2012) similar to Lazarsfeld’s. These studies have tried to extract what is qualitative by looking at the large number of empirical “qualitative” studies. Our novel strategy complements these endeavors by taking another approach and looking at the attempts to codify these practices in the form of a definition, as well as to a minor extent take Becker’s study as an exemplar of what qualitative researchers actually do, and what the characteristic of being ‘qualitative’ denotes and implies. We claim that qualitative researchers, if there is such a thing as “qualitative research,” should be able to codify their practices in a condensed, yet general way expressed in language.

Lingering problems of “generalizability” and “how many cases do I need” (Small 2009) are blocking advancement – in this line of work qualitative approaches are said to differ considerably from quantitative ones, while some of the former unsuccessfully mimic principles related to the latter (Small 2009). Additionally, quantitative researchers sometimes unfairly criticize the first based on their own quality criteria. Scholars like Goertz and Mahoney (2012) have successfully focused on the different norms and practices beyond what they argue are essentially two different cultures: those working with either qualitative or quantitative methods. Instead, similarly to Becker (2017) who has recently questioned the usefulness of the distinction between qualitative and quantitative research, we focus on similarities.

The current situation also impedes both students and researchers in focusing their studies and understanding each other’s work (Lazarsfeld and Barton 1982:239). A third consequence is providing an opening for critiques by scholars operating within different traditions (Valsiner 2000:101). A fourth issue is that the “implicit use of methods in qualitative research makes the field far less standardized than the quantitative paradigm” (Goertz and Mahoney 2012:9). Relatedly, the National Science Foundation in the US organized two workshops in 2004 and 2005 to address the scientific foundations of qualitative research involving strategies to improve it and to develop standards of evaluation in qualitative research. However, a specific focus on its distinguishing feature of being “qualitative” while being implicitly acknowledged, was discussed only briefly (for example, Best 2004).

In 2014 a theme issue was published in this journal on “Methods, Materials, and Meanings: Designing Cultural Analysis,” discussing central issues in (cultural) qualitative research (Berezin 2014; Biernacki 2014; Glaeser 2014; Lamont and Swidler 2014; Spillman 2014). We agree with many of the arguments put forward, such as the risk of methodological tribalism, and that we should not waste energy on debating methods separated from research questions. Nonetheless, a clarification of the relation to what is called “quantitative research” is of outmost importance to avoid misunderstandings and misguided debates between “qualitative” and “quantitative” researchers. Our strategy means that researchers, “qualitative” or “quantitative” they may be, in their actual practice may combine qualitative work and quantitative work.

In this article we accomplish three tasks. First, we systematically survey the literature for meanings of qualitative research by looking at how researchers have defined it. Drawing upon existing knowledge we find that the different meanings and ideas of qualitative research are not yet coherently integrated into one satisfactory definition. Next, we advance our contribution by offering a definition of qualitative research and illustrate its meaning and use partially by expanding on the brief example introduced earlier related to Becker’s work (1963). We offer a systematic analysis of central themes of what researchers consider to be the core of “qualitative,” regardless of style of work. These themes – which we summarize in terms of four keywords: distinction, process, closeness, improved understanding – constitute part of our literature review, in which each one appears, sometimes with others, but never all in the same definition. They serve as the foundation of our contribution. Our categories are overlapping. Their use is primarily to organize the large amount of definitions we have identified and analyzed, and not necessarily to draw a clear distinction between them. Finally, we continue the elaboration discussed above on the advantages of a clear definition of qualitative research.

### Methods

In a hermeneutic fashion we propose that there is something meaningful that deserves to be labelled “qualitative research” (Gadamer 1990). To approach the question “What is qualitative in qualitative research?” we have surveyed the literature. In conducting our survey we first traced the word’s etymology in dictionaries, encyclopedias, handbooks of the social sciences and of methods and textbooks, mainly in English, which is common to methodology courses. It should be noted that we have zoomed in on sociology and its literature. This discipline has been the site of the largest debate and development of methods that can be called “qualitative,” which suggests that this field should be examined in great detail.

In an ideal situation we should expect that one good definition, or at least some common ideas, would have emerged over the years. This common core of qualitative research should be so accepted that it would appear in at least some textbooks. Since this is not what we found, we decided to pursue an inductive approach to capture maximal variation in the field of qualitative research; we searched in a selection of handbooks, textbooks, book chapters, and books, to which we added the analysis of journal articles. Our sample comprises a total of 89 references.

#### Sampling

In practice we focused on the discipline that has had a clear discussion of methods, namely sociology. We also conducted a broad search in the JSTOR database to identify scholarly sociology articles published between 1998 and 2017 in English with a focus on defining or explaining qualitative research. We specifically zoom in on this time frame because we would have expect that this more mature period would have produced clear discussions on the meaning of qualitative research. To find these articles we combined a number of keywords to search the content and/or the title: *qualitative* (which was always included), *definition, empirical, research, methodology, studies, fieldwork, interview* and *observation*.

As a second phase of our research we searched within nine major sociological journals (*American Journal of Sociology*, *Sociological Theory*, *American Sociological Review*, *Contemporary Sociology*, *Sociological Forum*, *Sociological Theory*, *Qualitative Research*, *Qualitative Sociology* and *Qualitative Sociology Review*) for articles also published during the past 19 years (1998–2017) that had the term “qualitative” in the title and attempted to define qualitative research.

Lastly we picked two additional journals, *Qualitative Research* and *Qualitative Sociology*, in which we could expect to find texts addressing the notion of “qualitative.” From *Qualitative Research* we chose Volume 14, Issue 6, December 2014, and from *Qualitative Sociology* we chose Volume 36, Issue 2, June 2017. Within each of these we selected the first article; then we picked the second article of three prior issues. Again we went back another three issues and investigated article number three. Finally we went back another three issues and perused article number four. This selection criteria was used to get a manageable sample for the analysis.

#### Coding

The coding process of the 89 references we gathered in our selected review began soon after the first round of material was gathered, and we reduced the complexity created by our maximum variation sampling (Snow and Anderson 1993:22) to four different categories within which questions on the nature and properties of qualitative research were discussed. We call them: Qualitative and Quantitative Research, Qualitative Research, Fieldwork, and Grounded Theory. This – which may appear as an illogical grouping – merely reflects the “context” in which the matter of “qualitative” is discussed. If the selection process of the material – books and articles – was informed by pre-knowledge, we used an inductive strategy to code the material. When studying our material, we identified four central notions related to “qualitative” that appear in various combinations in the literature which indicate what is the core of qualitative research. We have labeled them: “distinctions”, “process,” “closeness,” and “improved understanding.” During the research process the categories and notions were improved, refined, changed, and reordered. The coding ended when a sense of saturation in the material arose. In the presentation below all quotations and references come from our empirical material of texts on qualitative research.

## Analysis – What is Qualitative Research?

In this section we describe the four categories we identified in the coding, how they differently discuss qualitative research, as well as their overall content. Some salient quotations are selected to represent the type of text sorted under each of the four categories. What we present are examples from the literature.

## Qualitative and Quantitative

This analytic category comprises quotations comparing qualitative and quantitative research, a distinction that is frequently used (Brown 2010:231); in effect this is a conceptual pair that structures the discussion and that may be associated with opposing interests. While the general goal of quantitative and qualitative *research* is the same – to understand the world better – their methodologies and focus in certain respects differ substantially (Becker 1966:55). Quantity refers to that property of something that can be determined by measurement. In a dictionary of Statistics and Methodology we find that “(a) When referring to *variables, ‘qualitative’ is another term for *categorical or *nominal. (b) When speaking of kinds of research, ‘qualitative’ refers to studies of subjects that are hard to quantify, such as art history. Qualitative research tends to be a residual category for almost any kind of non-quantitative research” (Stiles 1998:183). But it should be obvious that one could employ a quantitative approach when studying, for example, art history.

The same dictionary states that quantitative is “said of variables or research that can be handled numerically, usually (too sharply) contrasted with *qualitative variables and research” (Stiles 1998:184). From a qualitative perspective “quantitative research” is about numbers and counting, and from a quantitative perspective qualitative research is everything that is not about numbers. But this does not say much about what is “qualitative.” If we turn to encyclopedias we find that in the 1932 edition of the Encyclopedia of the Social Sciences there is no mention of “qualitative.” In the Encyclopedia from 1968 we can read:

Qualitative Analysis. For methods of obtaining, analyzing, and describing data, see [the various entries:] CONTENT ANALYSIS; COUNTED DATA; EVALUATION RESEARCH, FIELD WORK; GRAPHIC PRESENTATION; HISTORIOGRAPHY, especially the article on THE RHETORIC OF HISTORY; INTERVIEWING; OBSERVATION; PERSONALITY MEASUREMENT; PROJECTIVE METHODS; PSYCHOANALYSIS, article on EXPERIMENTAL METHODS; SURVEY ANALYSIS, TABULAR PRESENTATION; TYPOLOGIES. (Vol. 13:225)

Some, like Alford, divide researchers into methodologists or, in his words, “quantitative and qualitative specialists” (Alford 1998:12). Qualitative research uses a variety of methods, such as intensive interviews or in-depth analysis of historical materials, and it is concerned with a comprehensive account of some event or unit (King et al. 1994:4). Like quantitative research it can be utilized to study a variety of issues, but it tends to focus on meanings and motivations that underlie cultural symbols, personal experiences, phenomena and detailed understanding of processes in the social world. In short, qualitative research centers on understanding processes, experiences, and the meanings people assign to things (Kalof et al. 2008:79).

Others simply say that qualitative methods are inherently unscientific (Jovanović 2011:19). Hood, for instance, argues that words are intrinsically less precise than numbers, and that they are therefore more prone to subjective analysis, leading to biased results (Hood 2006:219). Qualitative methodologies have raised concerns over the limitations of quantitative templates (Brady et al. 2004:4). Scholars such as King et al. (1994), for instance, argue that non-statistical research can produce more reliable results if researchers pay attention to the rules of scientific inference commonly stated in quantitative research. Also, researchers such as Becker (1966:59; 1970:42–43) have asserted that, if conducted properly, qualitative research and in particular ethnographic field methods, can lead to more accurate results than quantitative studies, in particular, survey research and laboratory experiments.

Some researchers, such as Kalof, Dan, and Dietz (2008:79) claim that the boundaries between the two approaches are becoming blurred, and Small (2009) argues that currently much qualitative research (especially in North America) tries unsuccessfully and unnecessarily to emulate quantitative standards. For others, qualitative research tends to be more humanistic and discursive (King et al. 1994:4). Ragin (1994), and similarly also Becker, (1996:53), Marchel and Owens (2007:303) think that the main distinction between the two styles is overstated and does not rest on the simple dichotomy of “numbers versus words” (Ragin 1994:xii). Some claim that quantitative data can be utilized to discover associations, but in order to unveil cause and effect a complex research design involving the use of qualitative approaches needs to be devised (Gilbert 2009:35). Consequently, qualitative data are useful for understanding the nuances lying beyond those processes as they unfold (Gilbert 2009:35). Others contend that qualitative research is particularly well suited both to identify causality and to uncover fine descriptive distinctions (Fine and Hallett 2014; Lichterman and Isaac Reed 2014; Katz 2015).

There are other ways to separate these two traditions, including normative statements about what qualitative research should be (that is, better or worse than quantitative approaches, concerned with scientific approaches to societal change or vice versa; Snow and Morrill 1995; Denzin and Lincoln 2005), or whether it should develop falsifiable statements; Best 2004).

We propose that quantitative research is largely concerned with pre-determined variables (Small 2008); the analysis concerns the relations between variables. These categories are primarily not questioned in the study, only their frequency or degree, or the correlations between them (cf. Franzosi 2016). If a researcher studies wage differences between women and men, he or she works with given categories: *x* number of men are compared with *y* number of women, with a certain wage attributed to each person. The idea is not to move beyond the given categories of wage, men and women; they are the starting point as well as the end point, and undergo no “qualitative change.” Qualitative research, in contrast, investigates relations between categories that are themselves subject to change in the research process. Returning to Becker’s study (1963), we see that he questioned pre-dispositional theories of deviant behavior working with pre-determined variables such as an individual’s combination of personal qualities or emotional problems. His take, in contrast, was to understand marijuana consumption by developing “variables” as part of the investigation. Thereby he presented new variables, or as we would say today, theoretical concepts, but which are grounded in the empirical material.

### Qualitative Research

This category contains quotations that refer to descriptions of qualitative research without making comparisons with quantitative research. Researchers such as Denzin and Lincoln, who have written a series of influential handbooks on qualitative methods (1994; Denzin and Lincoln 2003; 2005), citing Nelson et al. (1992:4), argue that because qualitative research is “interdisciplinary, transdisciplinary, and sometimes counterdisciplinary” it is difficult to derive one single definition of it (Jovanović 2011:3). According to them, in fact, “the field” is “many things at the same time,” involving contradictions, tensions over its focus, methods, and how to derive interpretations and findings (2003: 11). Similarly, others, such as Flick (2007:ix–x) contend that agreeing on an accepted definition has increasingly become problematic, and that qualitative research has possibly matured different identities. However, Best holds that “the proliferation of many sorts of activities under the label of qualitative sociology threatens to confuse our discussions” (2004:54). Atkinson’s position is more definite: “the current state of qualitative research and research methods is confused” (2005:3–4).

Qualitative research is about interpretation (Blumer 1969; Strauss and Corbin 1998; Denzin and Lincoln 2003), or *Verstehen* [understanding] (Frankfort-Nachmias and Nachmias 1996). It is “multi-method,” involving the collection and use of a variety of empirical materials (Denzin and Lincoln 1998; Silverman 2013) and approaches (Silverman 2005; Flick 2007). It focuses not only on the objective nature of behavior but also on its subjective meanings: individuals’ own accounts of their attitudes, motivations, behavior (McIntyre 2005:127; Creswell 2009), events and situations (Bryman 1989) – what people say and do in specific places and institutions (Goodwin and Horowitz 2002:35–36) in social and temporal contexts (Morrill and Fine 1997). For this reason, following Weber ([1921-22] 1978), it can be described as an *interpretative science* (McIntyre 2005:127). But could quantitative research also be concerned with these questions? Also, as pointed out below, does all qualitative research focus on subjective meaning, as some scholars suggest?

Others also distinguish qualitative research by claiming that it collects data using a naturalistic approach (Denzin and Lincoln 2005:2; Creswell 2009), focusing on the meaning actors ascribe to their actions. But again, does all qualitative research need to be collected in situ? And does qualitative research have to be inherently concerned with meaning? Flick (2007), referring to Denzin and Lincoln (2005), mentions conversation analysis as an example of qualitative research that is not concerned with the meanings people bring to a situation, but rather with the formal organization of talk. Still others, such as Ragin (1994:85), note that qualitative research is often (especially early on in the project, we would add) less structured than other kinds of social research – a characteristic connected to its flexibility and that can lead both to potentially better, but also worse results. But is this not a feature of this type of research, rather than a defining description of its essence? Wouldn’t this comment also apply, albeit to varying degrees, to quantitative research?

In addition, Strauss (2003), along with others, such as Alvesson and Kärreman (2011:10–76), argue that qualitative researchers struggle to capture and represent complex phenomena partially because they tend to collect a large amount of data. While his analysis is correct at some points – “It is necessary to do detailed, intensive, microscopic examination of the data in order to bring out the amazing complexity of what lies in, behind, and beyond those data” (Strauss 2003:10) – much of his analysis concerns the supposed focus of qualitative research and its challenges, rather than exactly what it is about. But even in this instance we would make a weak case arguing that these are strictly the defining features of qualitative research. Some researchers seem to focus on the approach or the methods used, or even on the way material is analyzed. Several researchers stress the naturalistic assumption of investigating the world, suggesting that meaning and interpretation appear to be a core matter of qualitative research.

We can also see that in this category there is no consensus about specific qualitative methods nor about qualitative data. Many emphasize interpretation, but quantitative research, too, involves interpretation; the results of a regression analysis, for example, certainly have to be interpreted, and the form of meta-analysis that factor analysis provides indeed requires interpretation However, there is no interpretation of quantitative raw data, i.e., numbers in tables. One common thread is that qualitative researchers have to get to grips with their data in order to understand what is being studied in great detail, irrespective of the type of empirical material that is being analyzed. This observation is connected to the fact that qualitative researchers routinely make several adjustments of focus and research design as their studies progress, in many cases until the very end of the project (Kalof et al. 2008). If you, like Becker, do not start out with a detailed theory, adjustments such as the emergence and refinement of research questions will occur during the research process. We have thus found a number of useful reflections about qualitative research scattered across different sources, but none of them effectively describe the defining characteristics of this approach.

### Fieldwork

Although qualitative research does not appear to be defined in terms of a specific method, it is certainly common that fieldwork, i.e., research that entails that the researcher spends considerable time in the field that is studied and use the knowledge gained as data, is seen as emblematic of or even identical to qualitative research. But because we understand that fieldwork tends to focus primarily on the collection and analysis of qualitative data, we expected to find within it discussions on the meaning of “qualitative.” But, again, this was not the case.

Instead, we found material on the history of this approach (for example, Frankfort-Nachmias and Nachmias 1996; Atkinson et al. 2001), including how it has changed; for example, by adopting a more self-reflexive practice (Heyl 2001), as well as the different nomenclature that has been adopted, such as fieldwork, ethnography, qualitative research, naturalistic research, participant observation and so on (for example, Lofland et al. 2006; Gans 1999).

We retrieved definitions of ethnography, such as “the study of people acting in the natural courses of their daily lives,” involving a “resocialization of the researcher” (Emerson 1988:1) through intense immersion in others’ social worlds (see also examples in Hammersley 2018). This may be accomplished by direct observation and also participation (Neuman 2007:276), although others, such as Denzin (1970:185), have long recognized other types of observation, including non-participant (“fly on the wall”). In this category we have also isolated claims and opposing views, arguing that this type of research is distinguished primarily by where it is conducted (natural settings) (Hughes 1971:496), and how it is carried out (a variety of methods are applied) or, for some most importantly, by involving an active, empathetic immersion in those being studied (Emerson 1988:2). We also retrieved descriptions of the goals it attends in relation to how it is taught (understanding subjective meanings of the people studied, primarily develop theory, or contribute to social change) (see for example, Corte and Irwin 2017; Frankfort-Nachmias and Nachmias 1996:281; Trier-Bieniek 2012:639) by collecting the richest possible data (Lofland et al. 2006) to derive “thick descriptions” (Geertz 1973), and/or to aim at theoretical statements of general scope and applicability (for example, Emerson 1988; Fine 2003). We have identified guidelines on how to evaluate it (for example Becker 1996; Lamont 2004) and have retrieved instructions on how it should be conducted (for example, Lofland et al. 2006). For instance, analysis should take place while the data gathering unfolds (Emerson 1988; Hammersley and Atkinson 2007; Lofland et al. 2006), observations should be of long duration (Becker 1970:54; Goffman 1989), and data should be of high quantity (Becker 1970:52–53), as well as other questionable distinctions between fieldwork and other methods:

Field studies differ from other methods of research in that the researcher performs the task of selecting topics, decides what questions to ask, and forges interest *in the course of the research itself*. This is in sharp contrast to many ‘theory-driven’ and ‘hypothesis-testing’ methods. (Lofland and Lofland 1995:5)

But could not, for example, a strictly interview-based study be carried out with the same amount of flexibility, such as sequential interviewing (for example, Small 2009)? Once again, are quantitative approaches really as inflexible as some qualitative researchers think? Moreover, this category stresses the role of the actors’ meaning, which requires knowledge and close interaction with people, their practices and their lifeworld.

It is clear that field studies – which are seen by some as the “gold standard” of qualitative research – are nonetheless only one way of doing qualitative research. There are other methods, but it is not clear why some are more qualitative than others, or why they are better or worse. Fieldwork is characterized by interaction with the field (the material) and understanding of the phenomenon that is being studied. In Becker’s case, he had general experience from fields in which marihuana was used, based on which he did interviews with actual users in several fields.

### Grounded Theory

Another major category we identified in our sample is Grounded Theory. We found descriptions of it most clearly in Glaser and Strauss’ ([1967] 2010) original articulation, Strauss and Corbin (1998) and Charmaz (2006), as well as many other accounts of what it is for: generating and testing theory (Strauss 2003:xi). We identified explanations of how this task can be accomplished – such as through two main procedures: *constant comparison* and *theoretical sampling* (Emerson 1998:96), and how using it has helped researchers to “think differently” (for example, Strauss and Corbin 1998:1). We also read descriptions of its main traits, what it entails and fosters – for instance, an exceptional flexibility, an inductive approach (Strauss and Corbin 1998:31–33; 1990; Esterberg 2002:7), an ability to step back and critically analyze situations, recognize tendencies towards bias, think abstractly and be open to criticism, enhance sensitivity towards the words and actions of respondents, and develop a sense of absorption and devotion to the research process (Strauss and Corbin 1998:5–6). Accordingly, we identified discussions of the value of triangulating different methods (both using and not using grounded theory), including quantitative ones, and theories to achieve theoretical development (most comprehensively in Denzin 1970; Strauss and Corbin 1998; Timmermans and Tavory 2012). We have also located arguments about how its practice helps to systematize data collection, analysis and presentation of results (Glaser and Strauss [1967] 2010:16).

Grounded theory offers a systematic approach which requires researchers to get close to the field; closeness is a requirement of identifying questions and developing new concepts or making further distinctions with regard to old concepts. In contrast to other qualitative approaches, grounded theory emphasizes the detailed coding process, and the numerous fine-tuned distinctions that the researcher makes during the process. Within this category, too, we could not find a satisfying discussion of the meaning of qualitative research.

## Defining Qualitative Research

In sum, our analysis shows that some notions reappear in the discussion of qualitative research, such as understanding, interpretation, “getting close” and making distinctions. These notions capture aspects of what we think is “qualitative.” However, a comprehensive definition that is useful and that can further develop the field is lacking, and not even a clear picture of its essential elements appears. In other words no definition emerges from our data, and in our research process we have moved back and forth between our empirical data and the attempt to present a definition. Our concrete strategy, as stated above, is to relate qualitative and quantitative research, or more specifically, qualitative and quantitative work. We use an ideal-typical notion of quantitative research which relies on taken for granted and numbered variables. This means that the data consists of variables on different scales, such as ordinal, but frequently ratio and absolute scales, and the representation of the numbers to the variables, i.e. the justification of the assignment of numbers to object or phenomenon, are not questioned, though the validity may be questioned. In this section we return to the notion of quality and try to clarify it while presenting our contribution.

Broadly, research refers to the activity performed by people trained to obtain knowledge through systematic procedures. Notions such as “objectivity” and “reflexivity,” “systematic,” “theory,” “evidence” and “openness” are here taken for granted in any type of research. Next, building on our empirical analysis we explain the four notions that we have identified as central to qualitative work: distinctions, process, closeness, and improved understanding. In discussing them, ultimately in relation to one another, we make their meaning even more precise. Our idea, in short, is that only when these ideas that we present separately for analytic purposes are brought together can we speak of qualitative research.

### Distinctions

We believe that the possibility of making new distinctions is one the defining characteristics of qualitative research. It clearly sets it apart from quantitative analysis which works with taken-for-granted variables, albeit as mentioned, meta-analyses, for example, factor analysis may result in new variables. “Quality” refers essentially to distinctions, as already pointed out by Aristotle. He discusses the term “qualitative” commenting: “By a quality I mean that in virtue of which things are said to be qualified somehow” (Aristotle 1984:14). Quality is about what something is or has, which means that the distinction from its environment is crucial. We see qualitative research as a process in which significant new distinctions are made to the scholarly community; to make distinctions is a key aspect of obtaining new knowledge; a point, as we will see, that also has implications for “quantitative research.” The notion of being “significant” is paramount. New distinctions by themselves are not enough; just adding concepts only increases complexity without furthering our knowledge. The significance of new distinctions is judged against the communal knowledge of the research community. To enable this discussion and judgements central elements of rational discussion are required (cf. Habermas [1981] 1987; Davidsson [1988] 2001) to identify what is new and relevant scientific knowledge. Relatedly, Ragin alludes to the idea of new and useful knowledge at a more concrete level: “Qualitative methods are appropriate for in-depth examination of cases because they aid the identification of key features of cases. Most qualitative methods *enhance* data” (1994:79). When Becker (1963) studied deviant behavior and investigated how people became marihuana smokers, he made distinctions between the ways in which people learned how to smoke. This is a classic example of how the strategy of “getting close” to the material, for example the text, people or pictures that are subject to analysis, may enable researchers to obtain deeper insight and new knowledge by making distinctions – in this instance on the initial notion of learning how to smoke. Others have stressed the making of distinctions in relation to coding or theorizing. Emerson et al. (1995), for example, hold that “qualitative coding is a way of opening up avenues of inquiry,” meaning that the researcher identifies and develops concepts and analytic insights through close examination of and reflection on data (Emerson et al. 1995:151). Goodwin and Horowitz highlight making distinctions in relation to theory-building writing: “Close engagement with their cases typically requires qualitative researchers to adapt existing theories or to make new conceptual distinctions or theoretical arguments to accommodate new data” (2002: 37). In the ideal-typical quantitative research only existing and so to speak, given, variables would be used. If this is the case no new distinction are made. But, would not also many “quantitative” researchers make new distinctions?

### Process

Process does not merely suggest that research takes time. It mainly implies that qualitative new knowledge results from a process that involves several phases, and above all iteration. Qualitative research is about oscillation between theory and evidence, analysis and generating material, between *first-* and *second*-order constructs (Schütz 1962:59), between getting in contact with something, finding sources, becoming deeply familiar with a topic, and then distilling and communicating some of its essential features. The main point is that the categories that the researcher uses, and perhaps takes for granted at the beginning of the research process, usually undergo qualitative changes resulting from what is found. Becker describes how he tested hypotheses and let the jargon of the users develop into theoretical concepts. This happens over time while the study is being conducted, exemplifying what we mean by process.

In the research process, a pilot-study may be used to get a first glance of, for example, the field, how to approach it, and what methods can be used, after which the method and theory are chosen or refined before the main study begins. Thus, the empirical material is often central from the start of the project and frequently leads to adjustments by the researcher. Likewise, during the main study categories are not fixed; the empirical material is seen in light of the theory used, but it is also given the opportunity to kick back, thereby resisting attempts to apply theoretical straightjackets (Becker 1970:43). In this process, coding and analysis are interwoven, and thus are often important steps for getting closer to the phenomenon and deciding what to focus on next. Becker began his research by interviewing musicians close to him, then asking them to refer him to other musicians, and later on doubling his original sample of about 25 to include individuals in other professions (Becker 1973:46). Additionally, he made use of some participant observation, documents, and interviews with opiate users made available to him by colleagues. As his inductive theory of deviance evolved, Becker expanded his sample in order to fine tune it, and test the accuracy and generality of his hypotheses. In addition, he introduced a negative case and discussed the null hypothesis (1963:44). His phasic career model is thus based on a research design that embraces processual work. Typically, process means to move between “theory” and “material” but also to deal with negative cases, and Becker (1998) describes how discovering these negative cases impacted his research design and ultimately its findings.

Obviously, all research is process-oriented to some degree. The point is that the ideal-typical quantitative process does not imply change of the data, and iteration between data, evidence, hypotheses, empirical work, and theory. The data, quantified variables, are, in most cases fixed. Merging of data, which of course can be done in a quantitative research process, does not mean new data. New hypotheses are frequently tested, but the “raw data is often the “the same.” Obviously, over time new datasets are made available and put into use.

### Closeness

Another characteristic that is emphasized in our sample is that qualitative researchers – and in particular ethnographers – can, or as Goffman put it, ought to (1989), get closer to the phenomenon being studied and their data than quantitative researchers (for example, Silverman 2009:85). Put differently, essentially because of their methods qualitative researchers get into direct *close* contact with those being investigated and/or the material, such as texts, being analyzed. Becker started out his interview study, as we noted, by talking to those he knew in the field of music to get closer to the phenomenon he was studying. By conducting interviews he got even closer. Had he done more observations, he would undoubtedly have got even closer to the field.

Additionally, ethnographers’ design enables researchers to follow the field over time, and the research they do is almost by definition longitudinal, though the time in the field is studied obviously differs between studies. The general characteristic of closeness over time maximizes the chances of unexpected events, new data (related, for example, to archival research as additional sources, and for ethnography for situations not necessarily previously thought of as instrumental – what Mannay and Morgan (2015) term the “waiting field”), serendipity (Merton and Barber 2004; Åkerström 2013), and possibly reactivity, as well as the opportunity to observe disrupted patterns that translate into exemplars of negative cases. Two classic examples of this are Becker’s finding of what medical students call “crocks” (Becker et al. 1961:317), and Geertz’s (1973) study of “deep play” in Balinese society.

By getting and *staying* so close to their data – be it pictures, text or humans interacting (Becker was himself a musician) – for a long time, as the research progressively focuses, qualitative researchers are prompted to continually test their hunches, presuppositions and hypotheses. They test them against a reality that often (but certainly not always), and practically, as well as metaphorically, talks back, whether by validating them, or disqualifying their premises – correctly, as well as incorrectly (Fine 2003; Becker 1970). This testing nonetheless often leads to new directions for the research. Becker, for example, says that he was initially reading psychological theories, but when facing the data he develops a theory that looks at, you may say, everything but psychological dispositions to explain the use of marihuana. Especially researchers involved with ethnographic methods have a fairly unique opportunity to dig up and then test (in a circular, continuous and temporal way) new research questions and findings as the research progresses, and thereby to derive previously unimagined and uncharted distinctions by getting closer to the phenomenon under study.

Let us stress that getting close is by no means restricted to ethnography. The notion of hermeneutic circle and hermeneutics as a general way of understanding implies that we must get close to the details in order to get the big picture. This also means that qualitative researchers can literally also make use of details of pictures as evidence (cf. Harper 2002). Thus, researchers may get closer both when generating the material or when analyzing it.

Quantitative research, we maintain, in the ideal-typical representation cannot get closer to the data. The data is essentially numbers in tables making up the variables (Franzosi 2016:138). The data may originally have been “qualitative,” but once reduced to numbers there can only be a type of “hermeneutics” about what the number may stand for. The numbers themselves, however, are non-ambiguous. Thus, in quantitative research, interpretation, if done, is not about the data itself—the numbers—but what the numbers stand for. It follows that the interpretation is essentially done in a more “speculative” mode without direct empirical evidence (cf. Becker 2017).

### Improved Understanding

While distinction, process and getting closer refer to the qualitative work of the researcher, improved understanding refers to its conditions and outcome of this work. Understanding cuts deeper than explanation, which to some may mean a causally verified correlation between variables. The notion of explanation presupposes the notion of understanding since explanation does not include an idea of how knowledge is gained (Manicas 2006: 15). Understanding, we argue, is the core concept of what we call the outcome of the process when research has made use of all the other elements that were integrated in the research. Understanding, then, has a special status in qualitative research since it refers both to the conditions of knowledge and the outcome of the process. Understanding can to some extent be seen as the condition of explanation and occurs in a process of interpretation, which naturally refers to meaning (Gadamer 1990). It is fundamentally connected to knowing, and to the knowing of how to do things (Heidegger [1927] 2001). Conceptually the term hermeneutics is used to account for this process. Heidegger ties hermeneutics to human being and not possible to separate from the understanding of being (1988). Here we use it in a broader sense, and more connected to method in general (cf. Seiffert 1992). The abovementioned aspects – for example, “objectivity” and “reflexivity” – of the approach are conditions of *scientific* understanding. Understanding is the result of a circular process and means that the parts are understood in light of the whole, and vice versa. Understanding presupposes pre-understanding, or in other words, some knowledge of the phenomenon studied. The pre-understanding, even in the form of prejudices, are in qualitative research process, which we see as iterative, questioned, which gradually or suddenly change due to the iteration of data, evidence and concepts. However, qualitative research generates understanding in the iterative process when the researcher gets closer to the data, e.g., by going back and forth between field and analysis in a process that generates new data that changes the evidence, and, ultimately, the findings. Questioning, to ask questions, and put what one assumes—prejudices and presumption—in question, is central to understand something (Heidegger [1927] 2001; Gadamer 1990:368–384). We propose that this iterative process in which the process of understanding occurs is characteristic of qualitative research.

Improved understanding means that we obtain scientific knowledge of something that we as a scholarly community did not know before, or that we get to know something better. It means that we understand more about how parts are related to one another, and to other things we already understand (see also Fine and Hallett 2014). Understanding is an important condition for qualitative research. It is not enough to identify correlations, make distinctions, and work in a process in which one gets close to the field or phenomena. Understanding is accomplished when the elements are integrated in an iterative process.

It is, moreover, possible to understand many things, and researchers, just like children, may come to understand new things every day as they engage with the world. This subjective condition of understanding – namely, that a person gains a better understanding of something –is easily met. To be qualified as “scientific,” the understanding must be general and useful to many; it must be public. But even this generally accessible understanding is not enough in order to speak of “scientific understanding.” Though we as a collective can increase understanding of everything in virtually all potential directions as a result also of qualitative work, we refrain from this “objective” way of understanding, which has no means of discriminating between what we gain in understanding. Scientific understanding means that it is deemed relevant from the scientific horizon (compare Schütz 1962: 35–38, 46, 63), and that it rests on the pre-understanding that the scientists have and must have in order to understand. In other words, the understanding gained must be deemed useful by other researchers, so that they can build on it. We thus see understanding from a pragmatic, rather than a subjective or objective perspective. Improved understanding is related to the question(s) at hand. Understanding, in order to represent an improvement, must be an improvement in relation to the existing body of knowledge of the scientific community (James [1907] 1955). Scientific understanding is, by definition, collective, as expressed in Weber’s famous note on objectivity, namely that scientific work aims at truths “which … can claim, even for a Chinese, the validity appropriate to an empirical analysis” ([1904] 1949:59). By qualifying “improved understanding” we argue that it is a general defining characteristic of qualitative research. Becker‘s (1966) study and other research of deviant behavior increased our understanding of the social learning processes of how individuals start a behavior. And it also added new knowledge about the labeling of deviant behavior as a social process. Few studies, of course, make the same large contribution as Becker’s, but are nonetheless qualitative research.

Understanding in the phenomenological sense, which is a hallmark of qualitative research, we argue, requires meaning and this meaning is derived from the context, and above all the data being analyzed. The ideal-typical quantitative research operates with given variables with different numbers. This type of material is not enough to establish meaning at the level that truly justifies understanding. In other words, many social science explanations offer ideas about correlations or even causal relations, but this does not mean that the meaning at the level of the data analyzed, is understood. This leads us to say that there are indeed many explanations that meet the criteria of understanding, for example the explanation of how one becomes a marihuana smoker presented by Becker. However, we may also understand a phenomenon without explaining it, and we may have potential explanations, or better correlations, that are not really understood.

We may speak more generally of quantitative research and its data to clarify what we see as an important distinction. The “raw data” that quantitative research—as an idealtypical activity, refers to is not available for further analysis; the numbers, once created, are not to be questioned (Franzosi 2016: 138). If the researcher is to do “more” or “change” something, this will be done by conjectures based on theoretical knowledge or based on the researcher’s lifeworld. Both qualitative and quantitative research is based on the lifeworld, and all researchers use prejudices and pre-understanding in the research process. This idea is present in the works of Heidegger (2001) and Heisenberg (cited in Franzosi 2010:619). Qualitative research, as we argued, involves the interaction and questioning of concepts (theory), data, and evidence.

## Conclusion

Ragin (2004:22) points out that “a good definition of qualitative research should be inclusive and should emphasize its key strengths and features, not what it lacks (for example, the use of sophisticated quantitative techniques).” We define qualitative research as an iterative process in which improved understanding to the scientific community is achieved by making new significant distinctions resulting from getting closer to the phenomenon studied. Qualitative research, as defined here, is consequently a combination of two criteria: (i) how to do things –namely, generating and analyzing empirical material, in an iterative process in which one gets closer by making distinctions, and (ii) the outcome –improved understanding novel to the scholarly community. Is our definition applicable to our own study? In this study we have closely read the empirical material that we generated, and the novel distinction of the notion “qualitative research” is the outcome of an iterative process in which both deduction and induction were involved, in which we identified the categories that we analyzed. We thus claim to meet the first criteria, “how to do things.” The second criteria cannot be judged but in a partial way by us, namely that the “outcome” —in concrete form the definition-improves our understanding to others in the scientific community.

We have defined qualitative research, or qualitative scientific work, in relation to quantitative scientific work. Given this definition, qualitative research is about questioning the pre-given (taken for granted) variables, but it is thus also about making new distinctions of any type of phenomenon, for example, by coining new concepts, including the identification of new variables. This process, as we have discussed, is carried out in relation to empirical material, previous research, and thus in relation to theory. Theory and previous research cannot be escaped or bracketed. According to hermeneutic principles all scientific work is grounded in the lifeworld, and as social scientists we can thus never fully bracket our pre-understanding.

We have proposed that quantitative research, as an idealtype, is concerned with pre-determined variables (Small 2008). Variables are epistemically fixed, but can vary in terms of dimensions, such as frequency or number. Age is an example; as a variable it can take on different numbers. In relation to quantitative research, qualitative research does not reduce its material to number and variables. If this is done the process of comes to a halt, the researcher gets more distanced from her data, and it makes it no longer possible to make new distinctions that increase our understanding. We have above discussed the components of our definition in relation to quantitative research. Our conclusion is that in the research that is called quantitative there are frequent and necessary qualitative elements.

Further, comparative empirical research on researchers primarily working with ”quantitative” approaches and those working with ”qualitative” approaches, we propose, would perhaps show that there are many similarities in practices of these two approaches. This is not to deny dissimilarities, or the different epistemic and ontic presuppositions that may be more or less strongly associated with the two different strands (see Goertz and Mahoney 2012). Our point is nonetheless that prejudices and preconceptions about researchers are unproductive, and that as other researchers have argued, differences may be exaggerated (e.g., Becker 1996: 53, 2017; Marchel and Owens 2007:303; Ragin 1994), and that a qualitative dimension is present in both kinds of work.

Several things follow from our findings. The most important result is the relation to quantitative research. In our analysis we have separated qualitative research from quantitative research. The point is not to label individual researchers, methods, projects, or works as either “quantitative” or “qualitative.” By analyzing, i.e., taking apart, the notions of quantitative and qualitative, we hope to have shown the elements of qualitative research. Our definition captures the elements, and how they, when combined in practice, generate understanding. As many of the quotations we have used suggest, one conclusion of our study holds that qualitative approaches are not inherently connected with a specific method. Put differently, none of the methods that are frequently labelled “qualitative,” such as interviews or participant observation, are inherently “qualitative.” What matters, given our definition, is whether one works qualitatively or quantitatively in the research process, until the results are produced. Consequently, our analysis also suggests that those researchers working with what in the literature and in jargon is often called “quantitative research” are almost bound to make use of what we have identified as qualitative elements in any research project. Our findings also suggest that many” quantitative” researchers, at least to some extent, are engaged with qualitative work, such as when research questions are developed, variables are constructed and combined, and hypotheses are formulated. Furthermore, a research project may hover between “qualitative” and “quantitative” or start out as “qualitative” and later move into a “quantitative” (a distinct strategy that is not similar to “mixed methods” or just simply combining induction and deduction). More generally speaking, the categories of “qualitative” and “quantitative,” unfortunately, often cover up practices, and it may lead to “camps” of researchers opposing one another. For example, regardless of the researcher is primarily oriented to “quantitative” or “qualitative” research, the role of theory is neglected (cf. Swedberg 2017). Our results open up for an interaction not characterized by differences, but by different emphasis, and similarities.

Let us take two examples to briefly indicate how qualitative elements can fruitfully be combined with quantitative. Franzosi (2010) has discussed the relations between quantitative and qualitative approaches, and more specifically the relation between words and numbers. He analyzes texts and argues that scientific meaning cannot be reduced to numbers. Put differently, the meaning of the numbers is to be understood by what is taken for granted, and what is part of the lifeworld (Schütz 1962). Franzosi shows how one can go about using qualitative and quantitative methods and data to address scientific questions analyzing violence in Italy at the time when fascism was rising (1919–1922). Aspers (2006) studied the meaning of fashion photographers. He uses an empirical phenomenological approach, and establishes meaning at the level of actors. In a second step this meaning, and the different ideal-typical photographers constructed as a result of participant observation and interviews, are tested using quantitative data from a database; in the first phase to verify the different ideal-types, in the second phase to use these types to establish new knowledge about the types. In both of these cases—and more examples can be found—authors move from qualitative data and try to keep the meaning established when using the quantitative data.

A second main result of our study is that a definition, and we provided one, offers a way for research to clarify, and even evaluate, what is done. Hence, our definition can guide researchers and students, informing them on how to think about concrete research problems they face, and to show what it means to get closer in a process in which new distinctions are made. The definition can also be used to evaluate the results, given that it is a standard of evaluation (cf. Hammersley 2007), to see whether new distinctions are made and whether this improves our understanding of what is researched, in addition to the evaluation of how the research was conducted. By making what is qualitative research explicit it becomes easier to communicate findings, and it is thereby much harder to fly under the radar with substandard research since there are standards of evaluation which make it easier to separate “good” from “not so good” qualitative research.

To conclude, our analysis, which ends with a definition of qualitative research can thus both address the “internal” issues of what is qualitative research, and the “external” critiques that make it harder to do qualitative research, to which both pressure from quantitative methods and general changes in society contribute.
